# Exercise and survival benefit in cancer patients: evidence from a comprehensive meta-analysis

**DOI:** 10.1007/s11357-025-01647-0

**Published:** 2025-04-12

**Authors:** Zoltan Ungvari, Mónika Fekete, Péter Varga, Gyöngyi Munkácsy, János Tibor Fekete, Andrea Lehoczki, Annamaria Buda, Csaba Kiss, Anna Ungvari, Balázs Győrffy

**Affiliations:** 1 Vascular Cognitive Impairment, Neurodegeneration and Healthy Brain Aging Program, Department of Neurosurgery, University of Oklahoma Health Sciences Center, Oklahoma City, OK USA; 2 Stephenson Cancer Center, University of Oklahoma, Oklahoma City, OK USA; 3 Oklahoma Center for Geroscience and Healthy Brain Aging, University of Oklahoma Health Sciences Center, Oklahoma City, OK USA; 4 Department of Health Promotion Sciences, College of Public Health, University of Oklahoma Health Sciences Center, Oklahoma City, OK USA; 5 International Training Program in Geroscience, Doctoral College, Health Sciences Division/Institute of Preventive Medicine and Public Health, Semmelweis University, Budapest, Hungary; 6 Institute of Preventive Medicine and Public Health, Semmelweis University, Semmelweis University, Budapest, Hungary; 7 Jozsef Fodor Center for Prevention and Healthy Aging, Semmelweis University, Budapest, Hungary; 8 Doctoral College, Health Sciences Division, Semmelweis University, Budapest, Hungary; 9 Dept. Of Bioinformatics, Semmelweis University, H- 1094 Budapest, Hungary; 10 Cancer Biomarker Research Group, Institute of Molecular Life Sciences, HUN-REN Research Centre for Natural Sciences, H- 1117 Budapest, Hungary; 11 Dept. Of Biophysics, Medical School, University of Pecs, H- 7624 Pecs, Hungary

**Keywords:** Physical activity, Survival, Breast cancer, Lung cancer, Prostate cancer, Colorectal cancer, Skin cancer

## Abstract

Cancer remains a major global health challenge, and growing evidence suggests that physical activity is a key modifiable factor that may improve survival outcomes in cancer patients. However, a comprehensive, large-scale synthesis of the effects of post-diagnosis physical activity across multiple cancer types remains lacking. This meta-analysis aims to systematically evaluate the association between physical activity and survival in patients diagnosed with breast, lung, prostate, colorectal, and skin cancers. We conducted a comprehensive search in PubMed, Web of Science, Scopus, and Cochrane Library for studies on physical activity and cancer survival. Eligible studies (January 2000–November 2024) included adults (≥ 18 years) with breast, lung, prostate, colorectal, or skin cancer. Only prospective cohort and case–control studies reporting hazard ratios (HRs) with 95% confidence intervals (CIs) for overall or cancer-specific mortality were included, with a minimum sample size of 100 and at least six months of follow-up. Meta-analysis was performed using metaanalysisonline.com, applying random-effects models and assessing heterogeneity via the I^2^ statistic. Sensitivity analyses and publication bias (Egger’s test, funnel plots) were evaluated. The meta-analysis included 151 cohorts with almost 1.5 million cancer patients. Post-diagnosis physical activity was associated with significantly lower cancer-specific mortality across all five cancer types. The greatest benefit was observed in breast cancer, with a pooled hazard ratio (HR) of 0.69 (95% CI: 0.63–0.75), followed by prostate cancer (HR: 0.73, 95% CI: 0.62–0.87). Lung cancer patients who engaged in physical activity had a 24% lower risk of cancer-specific death (HR: 0.76, 95% CI: 0.69–0.84), while colorectal cancer patients experienced a similar benefit (HR: 0.71, 95% CI: 0.63–0.80). In skin cancer, physical activity was associated with a non-significant reduction in mortality (HR: 0.86, 95% CI: 0.71–1.05). These findings provide robust evidence supporting the survival benefits of post-diagnosis physical activity in cancer patients, particularly for breast, prostate, lung, and colorectal cancers. The results underscore the potential for incorporating structured physical activity interventions into oncological care to improve long-term patient outcomes.

## Introduction

Cancer is one of the leading causes of morbidity and mortality worldwide [1–4], imposing a substantial burden on individuals, healthcare systems, and societies. According to the International Agency for Research on Cancer (IARC), an estimated 20 million new cancer cases and 9.7 million cancer-related deaths occurred globally in 2022, with projections indicating a 77% increase in incidence by 2050 [4–6]. Despite remarkable advancements in cancer detection, treatment, and supportive care, survival outcomes remain suboptimal for many patients, necessitating additional strategies to enhance prognosis and quality of life [7–10]. A significant proportion of these cases occur in older adults, as cancer is fundamentally an age-related disease, whose pathogenesis involve cellular and molecular mechanisms of aging[11]. The intersection of aging and cancer underscores the need for strategies that improve cancer survival by targeting the underlying biological mechanisms of aging[11].

Growing evidence suggests that physical activity may play a critical role in modulating these aging-related processes and improving cancer survival [12–22]. Beyond its well-documented benefits for cardiovascular and metabolic health, physical activity exerts profound anti-aging effects at the cellular and molecular levels [23–32]. Regular exercise has been shown to reduce chronic inflammation [33], prevent cellular senescence [34], enhance mitochondrial function [31, 35], maintain genomic stability[25, 36], and improve immune surveillance[37]— mechanisms that are intricately linked to both aging and cancer progression[38–41]. By interfering with these fundamental drivers of aging, physical activity may slow cancer progression and improve survival outcomes.

Despite the growing recognition of exercise as a key modifiable factor in both healthy aging and cancer prognosis, no comprehensive synthesis has systematically evaluated the impact of post-diagnosis physical activity across multiple age-related cancers. While individual studies and smaller meta-analyses have explored this relationship in specific cancer types[42–45], a large-scale, cross-cancer analysis is needed to establish the broader implications of physical activity in oncologic care.

Given the increasing recognition of exercise as a potential adjunct to conventional cancer therapies [46, 47], a rigorous evaluation of its impact on survival across different malignancies is warranted. This meta-analysis aims to systematically assess the association between post-diagnosis physical activity and survival outcomes in patients with breast, lung, prostate, colorectal, and skin cancers. By synthesizing data from a large body of evidence, this study seeks to provide clinically relevant insights that may inform future guidelines and interventions promoting physical activity in cancer care.

## Methods

We aimed to assess the influence of physical activity on survival outcomes in individuals diagnosed with five prevalent cancer types: breast, lung, prostate, colorectal, and skin cancer. Our primary focus was on overall mortality and cancer-specific mortality. To ensure a systematic and objective synthesis of the available evidence, we employed a rigorous methodological approach designed to identify, analyze, and integrate relevant studies.

### Search strategy

We conducted a comprehensive literature search across four widely used scientific databases to identify pertinent studies: PubMed, Web of Science, Scopus, and the Cochrane Library. The search encompassed publications from the year 2000 up to November 30, 2024. To maximize the retrieval of relevant studies, we developed search queries that combined disease-specific keywords with terms related to physical activity and survival outcomes. Finally, we also collected studies from previously published meta analyses [12–22].

For each cancer type, we applied the following search terms:

- Breast cancer: "breast cancer" AND "physical activity"; "exercise AND breast cancer survival"; "physical activity AND breast cancer recurrence"; "prognosis AND breast cancer AND exercise"
- Lung cancer: "lung cancer" AND "physical activity"; "exercise AND lung cancer survival"; "physical activity AND lung cancer recurrence"; "prognosis AND lung cancer AND exercise"
- Prostate cancer: "prostate cancer" AND "physical activity"; "exercise AND prostate cancer survival"; "physical activity AND prostate cancer recurrence"; "prognosis AND prostate cancer AND exercise"
- Colorectal cancer: "colorectal cancer" AND "physical activity"; "exercise AND colorectal cancer survival"; "physical activity AND colorectal cancer recurrence"; "prognosis AND colorectal cancer AND exercise"
- Skin cancer: "skin cancer" AND "physical activity"; "exercise AND skin cancer survival"; "physical activity AND skin cancer recurrence"; "prognosis AND skin cancer AND exercise"

**Fig. 1:**
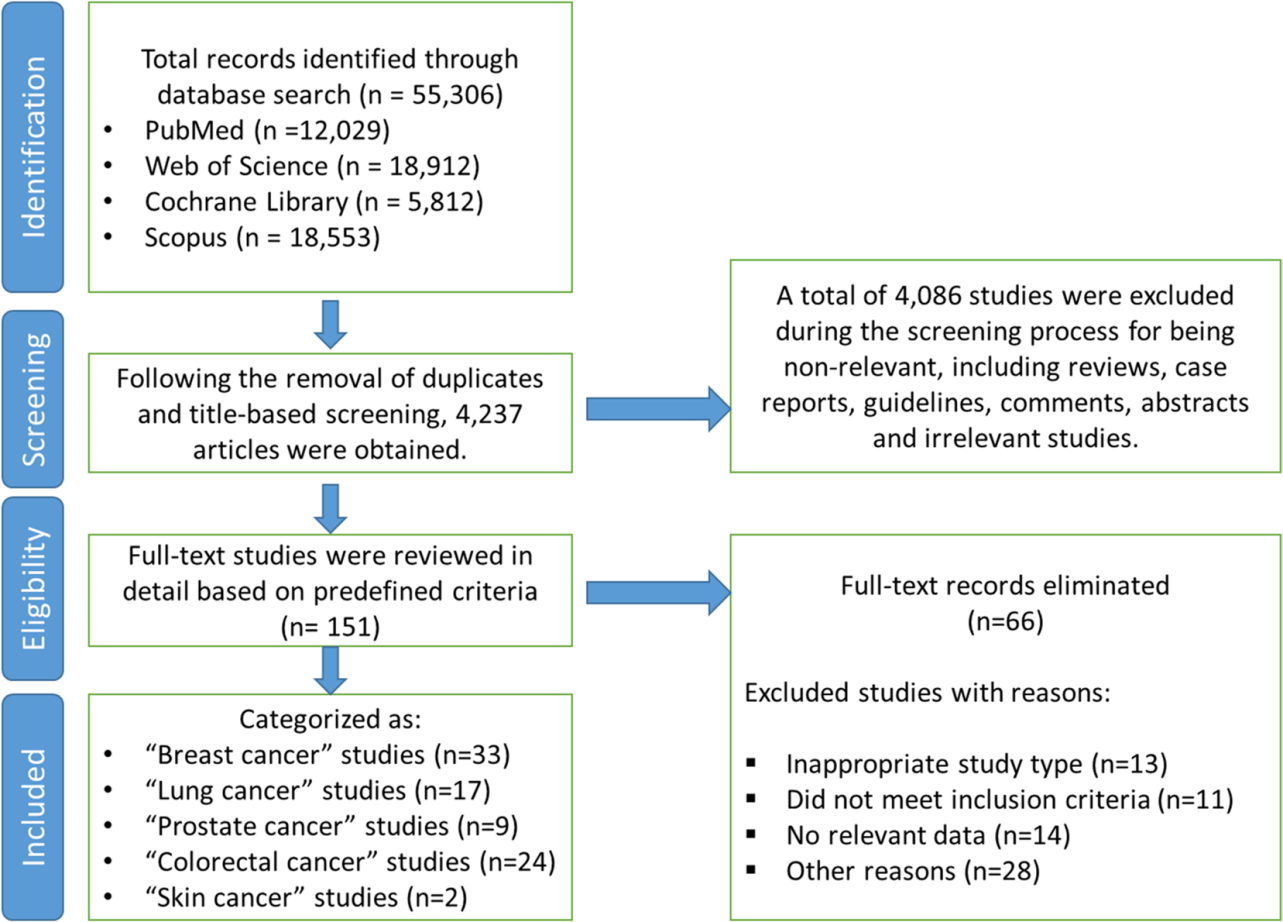
The flowchart illustrates the systematic selection process of studies examining the association between physical activity and survival of cancer patients

We conducted searches in the titles, abstracts, and keyword fields of each database. To refine our dataset, we excluded non-primary research articles such as editorials, commentaries, conference abstracts, and review papers. Additionally, we manually screened the reference lists of identified studies to capture any additional relevant publications. Figure 1 presents the study selection process in a flowchart.

Due to heterogeneity across studies, physical activity was defined based on each study’s operationalization—typically including self-reported duration, frequency, or intensity of exercise, or MET-hours/week. For the purposes of this meta-analysis, we extracted the hazard ratios comparing the highest versus lowest categories of post-diagnosis physical activity, as defined in each publication. Because the specific units and thresholds varied, we were unable to perform a dose–response meta-analysis.

To ensure methodological rigor, we applied predefined inclusion criteria. We considered studies eligible if they met the following conditions:

1. **Study design**: Prospective cohort studies and case–control studies were included.
2. **Participants**: Adults aged 18 years or older with a diagnosis of breast, lung, prostate, colorectal, or skin cancer.
3. **Outcomes**: Studies had to report either overall mortality or cancer-specific mortality.
4. **Sample size**: A minimum of 100 participants was required to enhance statistical reliability.
5. **Follow-up period**: Studies with at least six months of follow-up were considered.
6. **Reported measures**: Studies needed to provide hazard ratios (HRs) with 95% confidence intervals (CIs) as measures of association.

We excluded studies that employed cross-sectional designs or failed to report survival outcomes as primary endpoints.

### Ethical considerations

Since this study was a meta-analysis that synthesized previously published research, we did not collect any individual patient data. Consequently, formal ethical approval was not required. However, we adhered to the Preferred Reporting Items for Systematic Reviews and Meta-Analyses (PRISMA) guidelines to ensure transparency and reproducibility.

### Statistical analysis

All statistical evaluations were performed utilizing the web-based software MetaAnalysisOnline.com [48]. Summary risk measures, including hazard ratios (HRs) and their associated 95% confidence intervals (CIs), were computed employing a random-effects model. This approach accounts for inter-study variability, enhancing the applicability of the results to broader populations. Risk ratios were estimated using hazard ratios, as these measures converge when event rates are low, and baseline risks are close to one [49]. To aid in the visualization of data and to emphasize variability, forest plots were generated to present findings from individual investigations alongside the combined summary estimate.

Heterogeneity across the included studies was assessed using Cochran’s Q test and the I^2^ statistic. Cochran’s Q test, which follows a chi-square distribution, was utilized to determine whether the observed differences in effect sizes were greater than those expected by random variation. The I^2^ statistic was employed to quantify the proportion of total variability attributable to genuine differences between studies as opposed to random error.

Subgroup analyses were carried out independently for all-cause mortality and cancer-specific mortality. For each subgroup, pooled effect measures were calculated, and heterogeneity was assessed using the same statistical approaches as in the primary analysis.

### Evaluation of publication bias

Potential publication bias was investigated using both graphical and analytical techniques. Funnel plots, which graph effect sizes against their precision measures, were visually examined for asymmetry, a potential indicator of publication bias. Furthermore, Egger’s regression test was performed to quantitatively evaluate the association between effect sizes and their standard errors, offering additional evidence regarding the presence of bias.

## Results

### Breast cancer

**Fig. 2:**
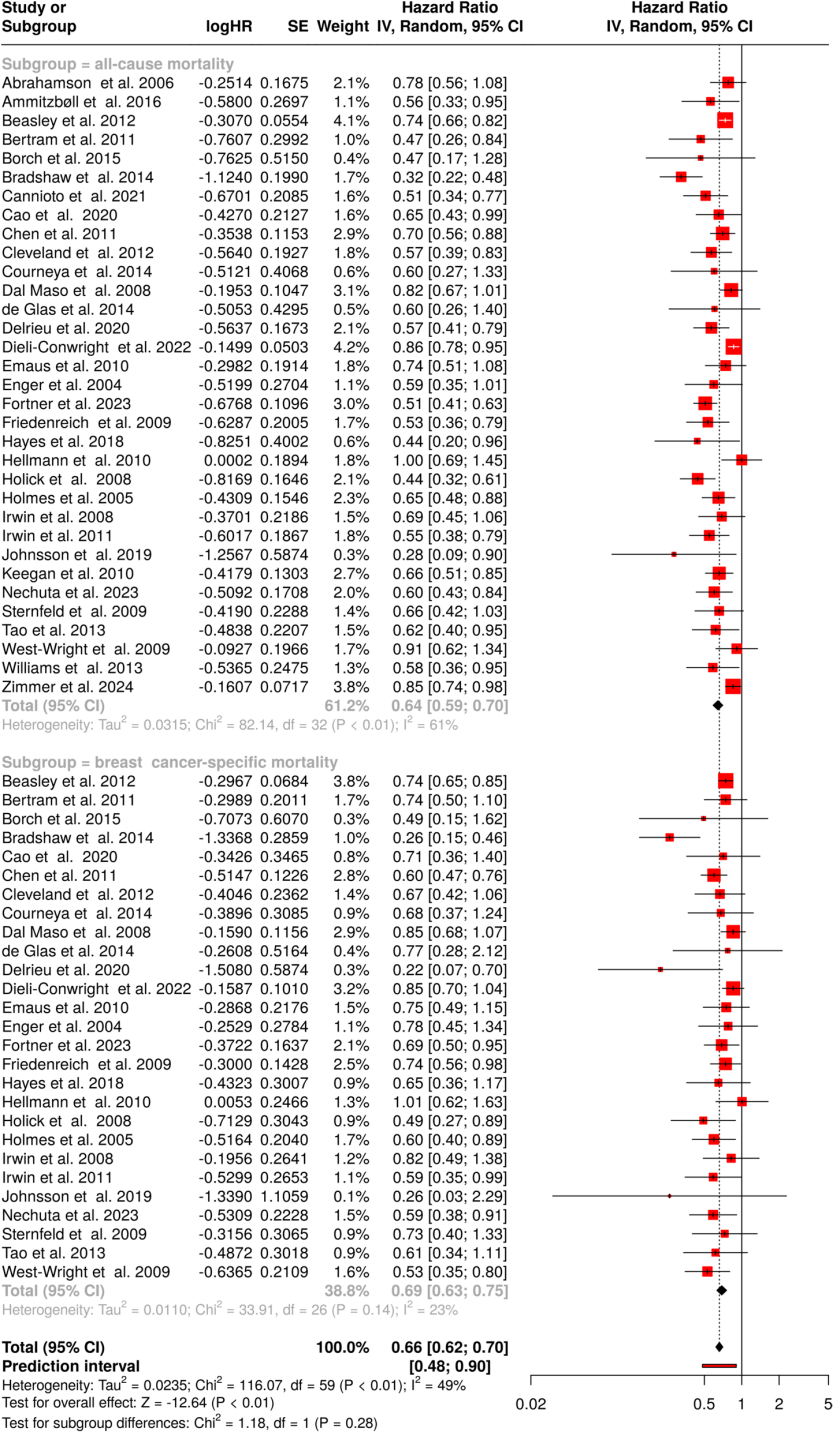
Meta-analysis of the association between physical activity and survival of breast cancer patients. The forest plot presents the pooled hazard ratios (HRs) for the association of physical activity with all-cause survival (top) and breast cancer-specific survival (bottom). Each study is represented by a red square, with the size proportional to its weight in the meta-analysis. Horizontal lines indicate 95% confidence intervals (CIs). The pooled HRs, derived from random-effects models, suggest a significant inverse association between physical activity and mortality of breast cancer patients (pooled HR: 0.66, 95% CI: 0.62–0.70). The analysis demonstrates moderate heterogeneity across studies (I^2^ = 49%, *p* < 0.01). Abbreviations:* CI*: confidence interval;* HR*:hazard ratio;* IV*: inverse variance; *SE*: standard error

This meta-analysis included 60 breast cohorts to assess the impact of physical activity on survival outcomes in breast cancer patients. The analysis was stratified to evaluate all-cause survival, breast cancer-specific survival, and their combined effect. For overall survival, 33 analyses [43, 50–81] were included (Fig. 2**,** upper panel). Physical activity was associated with a significantly lower risk of all-cause mortality among breast cancer patients, as evidenced by a pooled hazard estimate of 0.64, with a CI ranging from 0.59 to 0.70. The I^2^ statistic suggested that 61% of the observed differences were attributable to true heterogeneity rather than random variation, suggesting that factors such as study design, population characteristics, or physical activity assessment methods may have influenced the results (*p* < 0.01). For breast cancer-specific survival, a total of 27 studies were analyzed, as depicted in the lower panel of Fig. 2. The estimate indicated a statistically significant reduction in the risk of breast cancer-related death among physically active individuals, with an HR of 0.69 and a 95% CI of 0.63 to 0.75. Unlike the analysis of all-cause survival, this subgroup exhibited no significant heterogeneity, implying that the effect of physical activity on breast cancer-specific survival was consistent across studies (I^2^ = 23%, *p* = 0.14). When assessing the combined impact of physical activity on both all-cause and breast cancer-specific survival, the pooled HR was 0.66, with a CI spanning 0.62 to 0.70. The I^2^ value of 49% indicated that nearly half of the variability between studies could be attributed to actual differences rather than chance (*p* < 0.01).

**Fig. 3:**
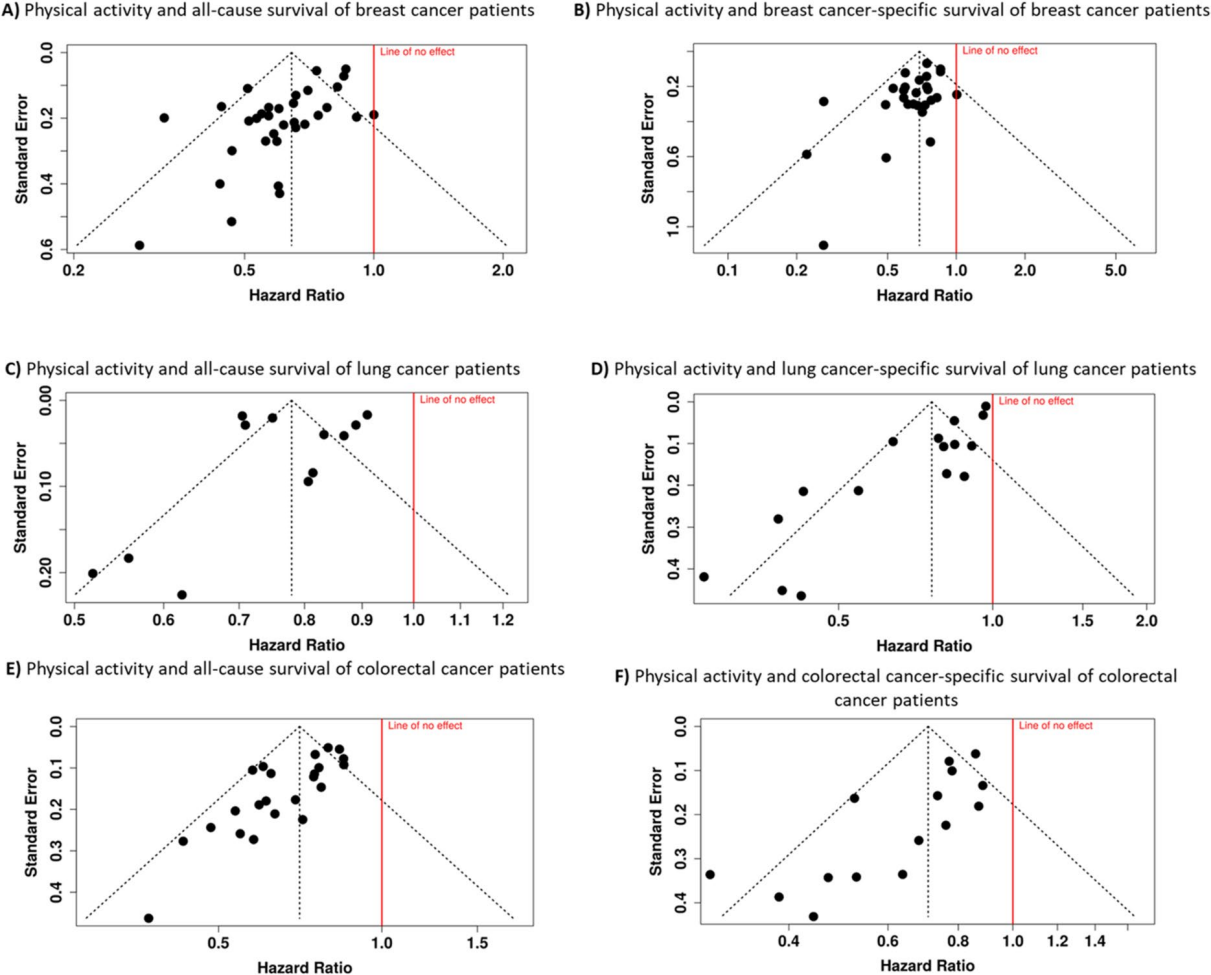
Funnel plots illustrating the relationship between hazard ratios (HRs) and standard errors for the association between physical activity and all-cause (**A, C, E**), and cancer-specific survival (**B, D, F**) in cancer patients. Breast cancer (**A, B**), lung cancer (**C, D**) and colorectal cancer studies (**E, F**) are illustrated. The shape and symmetry of the funnel plots can offer insights into potential publication bias, with asymmetrical plots indicating the possibility of selective reporting or publication of the studies with certain outcomes

To evaluate potential publication bias, funnel plots were constructed for both survival outcomes. The asymmetry observed in the funnel plots suggested the possibility of bias. For all-cause survival (Fig. 3A), Egger’s test indicated a publication bias, with an intercept of − 1.75, a CI from − 2.54 to − 0.95 (t = − 4.311, *p* < 0.001). Similarly, for breast cancer-specific survival (Fig. 3B), Egger’s regression analysis yielded an intercept of − 1.02, with a CI between − 1.76 and − 0.27 (t = − 2.667, *p* = 0.013), confirming significant asymmetry. These findings suggest that smaller studies with null or unfavorable results may be underrepresented in the literature, potentially influencing the overall effect estimates.

### Lung cancer

**Fig. 4:**
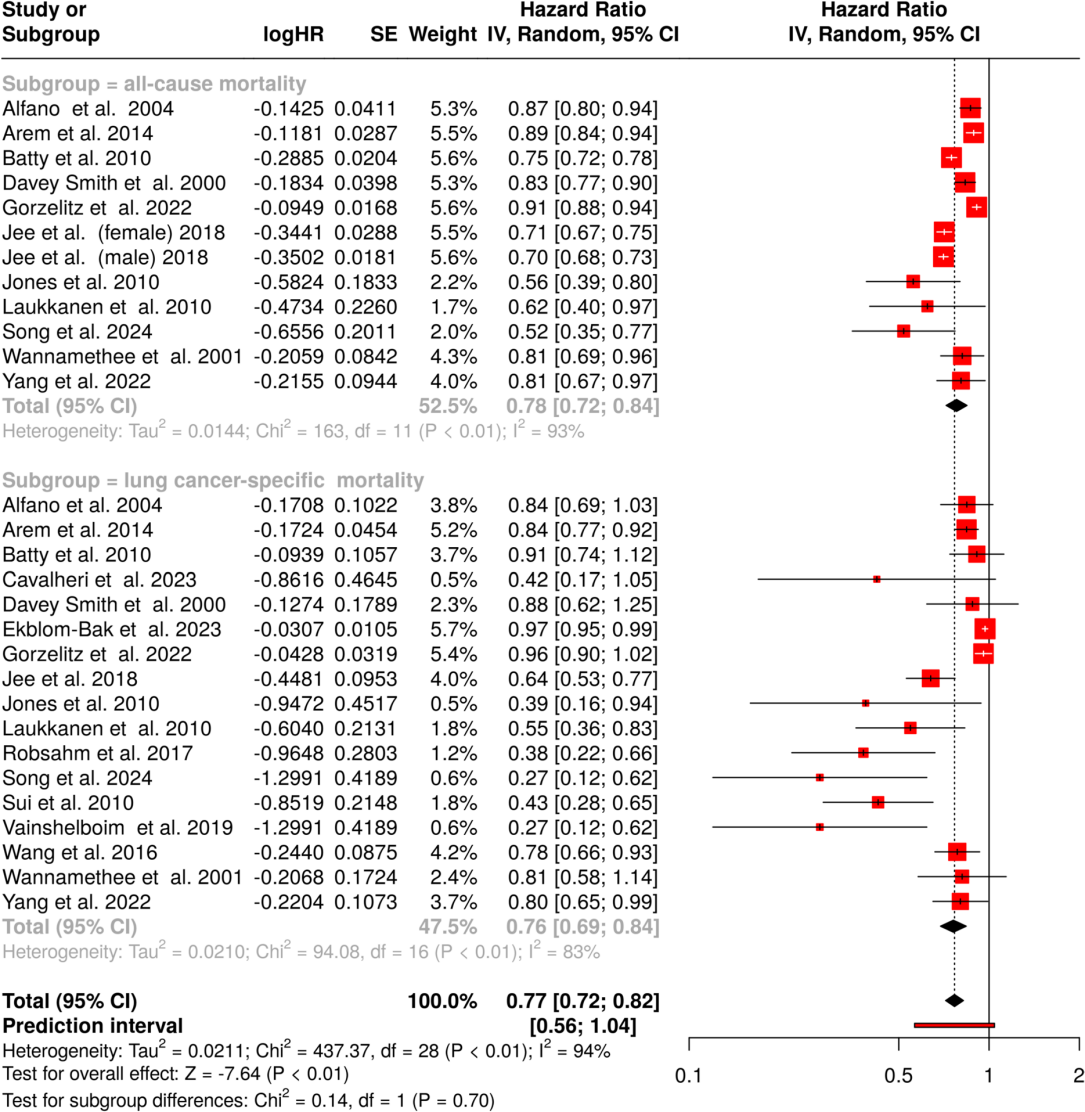
The forest plot summarizes the association between physical activity and all-cause (top) and lung cancer-specific mortality (bottom). Each study is represented by a red square, with its size reflecting the study’s weight in the meta-analysis. Horizontal lines indicate 95% confidence intervals (CIs). The results indicate a significant reduction in both all-cause and lung cancer-specific mortality among physically active individuals (pooled HR: 0.77, 95% CI: 0.72–0.82). Abbreviations:* CI*: confidence interval;* HR*: hazard ratio;* IV*: inverse variance;* SE*: standard error

A total of 29 cohorts were included in this meta-analysis to evaluate the relationship between physical activity and survival outcomes in lung cancer patients [82–98]. Analyses were conducted separately for all-cause and lung cancer-specific survival, as well as in a combined model to assess the overall impact (Fig. 4). The association between physical activity and all-cause survival was examined in 12 studies. The findings demonstrated a statistically significant association between higher physical activity levels and improved overall survival, with a 22% reduction in the risk of death from any cause (HR: 0.78, 95% CI: 0.72—0.84). However, substantial heterogeneity was detected (*p* < 0.01), with an I^2^ = 93%, indicating that nearly all the heterogeneity between studies was due to differences in study characteristics rather than chance. For the investigation of lung cancer-specific survival, 17 studies were analyzed. This analysis revealed a significant reduction in the risk of lung cancer-related death among physically active individuals, with a summary estimate indicating a 24% lower mortality rate (HR: 0.76, 95% CI: 0.69—0.84). Similar to all-cause survival, substantial heterogeneity was observed (*p* < 0.01), suggesting notable heterogeneity across studies in both effect size and direction. The I^2^ = 83% indicated that the majority of the observed differences stemmed from true heterogeneity rather than random variation. When evaluating the combined impact of physical activity on both survival outcomes, a statistically significant protective effect was confirmed, with an overall mortality reduction of 23% (HR: 0.77, 95% CI: 0.72—0.82). However, as with the individual analyses, the presence of significant heterogeneity (*p* < 0.01) and an I^2^ = 94% underscore the influence of study differences on the pooled estimates.

To assess potential publication bias, funnel plots were generated for both outcomes. The funnel plot for all-cause survival showed no strong asymmetry, suggesting the absence of publication bias (Fig. 3C). This finding was supported by Egger’s test, which yielded a non-significant result (intercept = − 0.89, 95% CI: − 4.76—2.99, t = − 0.448, *p* = 0.664), reinforcing the assumption that smaller studies with null or unfavorable results were not systematically excluded from the literature. In contrast, the funnel plot for lung cancer-specific survival suggested possible publication bias, as indicated by its asymmetrical distribution (Fig. 3D). Egger’s regression analysis confirmed the presence of bias, with a highly significant result (intercept = − 2.45, 95% CI: − 3.12—− 1.79, t = − 7.254, *p* < 0.001). This suggests that studies reporting smaller or non-significant associations may be underrepresented in the available literature, potentially influencing the estimated effect size.

### Prostate cancer

**Fig. 5:**
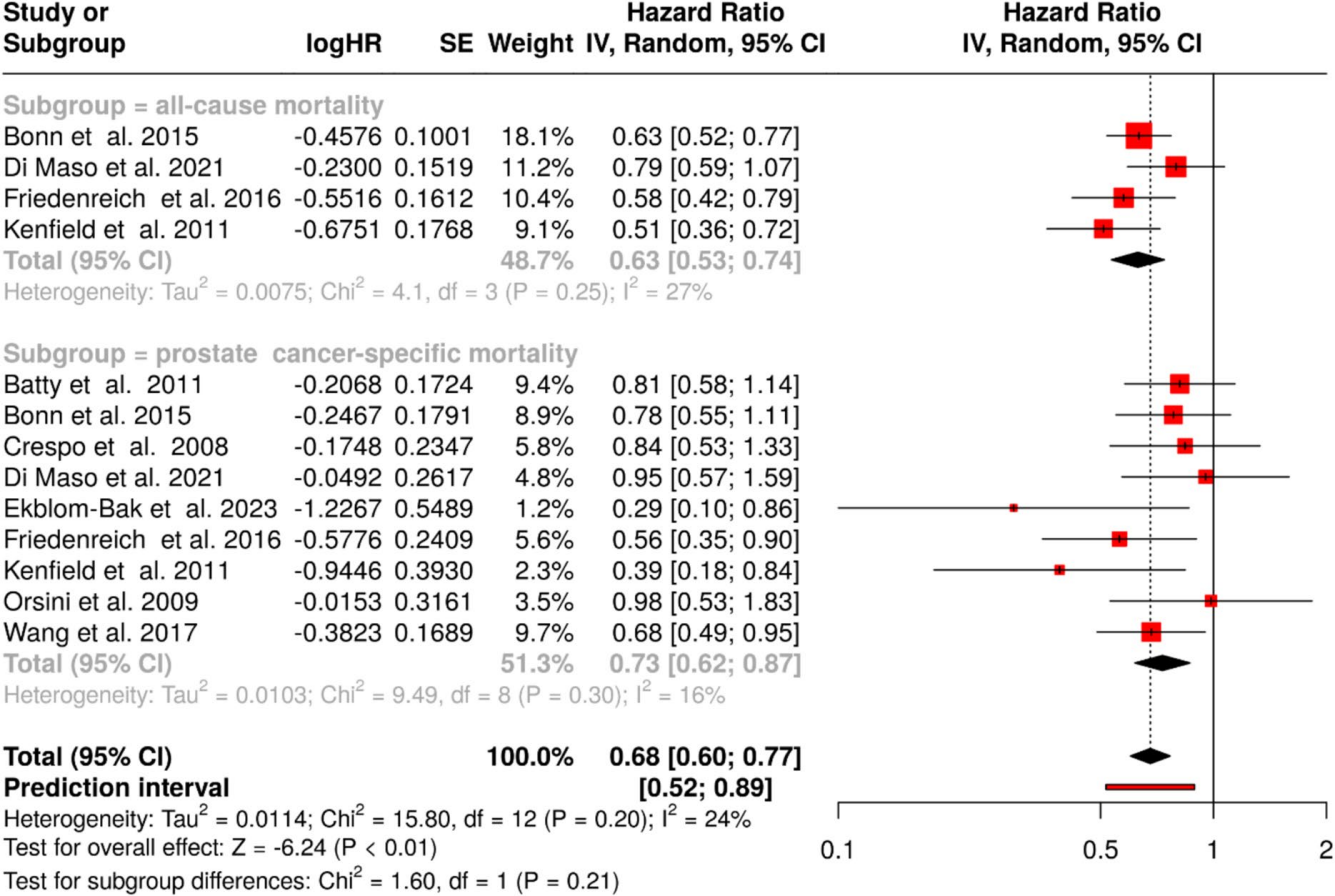
Meta-analysis of the association between physical activity and mortality of prostate cancer patients. The analysis stratified outcomes into all-cause survival (n = 4 studies) and prostate cancer-specific survival (n = 9 studies). The diamond represents the pooled effect size for each subgroup and overall effect, with values less than 1.0 indicating reduced mortality risk with physical activity (pooled hazard ratio (HR): 0.68, 95% confidence interval (CI): 0.6–0.77). Low between-study heterogeneity was observed in both subgroups (I^2^ = 27% and I^2^ = 16%, respectively) and in the pooled analysis (I^2^ = 24%, *p* = 0.20). Abbreviations:* CI*: confidence interval;* HR*: hazard ratio;* IV*: inverse variance;* SE*: standard error

Our meta-analysis examined the relationship between physical activity and survival outcomes in prostate cancer patients, encompassing 13 cohorts in total [98–106]. The analysis was stratified into two primary outcomes: all-cause survival and prostate cancer-specific survival. For all-cause survival, analysis of four studies demonstrated an even more pronounced benefit, showing a 37% reduction in all-cause mortality among physically active patients, with a pooled HR of 0.63 (95% CI: 0.53—0.74). These results exhibited minimal heterogeneity, indicating uniformity in the observed protective effect (I^2^ = 27%, *p* = 0.25) (Fig. 5**,** upper panel). The evaluation of prostate cancer-specific survival included nine studies (Fig. 5, lower panel). The analysis revealed that physical activity was associated with a 27% reduction in prostate cancer-specific mortality, with an HR of 0.73 (95% CI: 0.62—0.87). Similar to the all-cause findings, the absence of substantial heterogeneity in these findings suggests consistency in both the magnitude and direction of the protective effect across studies (I^2^ = 16%, *p* = 0.30). Pooled analysis demonstrated a robust protective effect of physical activity, yielding a pooled HR of 0.68 (95% CI: 0.60—0.77). This finding suggests that physical activity is associated with a 32% reduction in mortality risk across both outcome measures. The consistency of these results is supported by the minimal variability observed across studies (I^2^ = 24%, *p* = 0.20).

Given the limited number of studies, we assessed publication bias using a single funnel plot that included both survival outcomes. The funnel plot did not suggest any apparent publication bias (data not shown). This finding was further corroborated by Egger’s test, which revealed no significant asymmetry (intercept: − 0.24, 95% CI: − 1.98—1.5, t = − 0.268, *p* = 0.793), suggesting the robustness of our results in the absence of a potential publication bias.

### Colorectal cancer

**Fig. 6:**
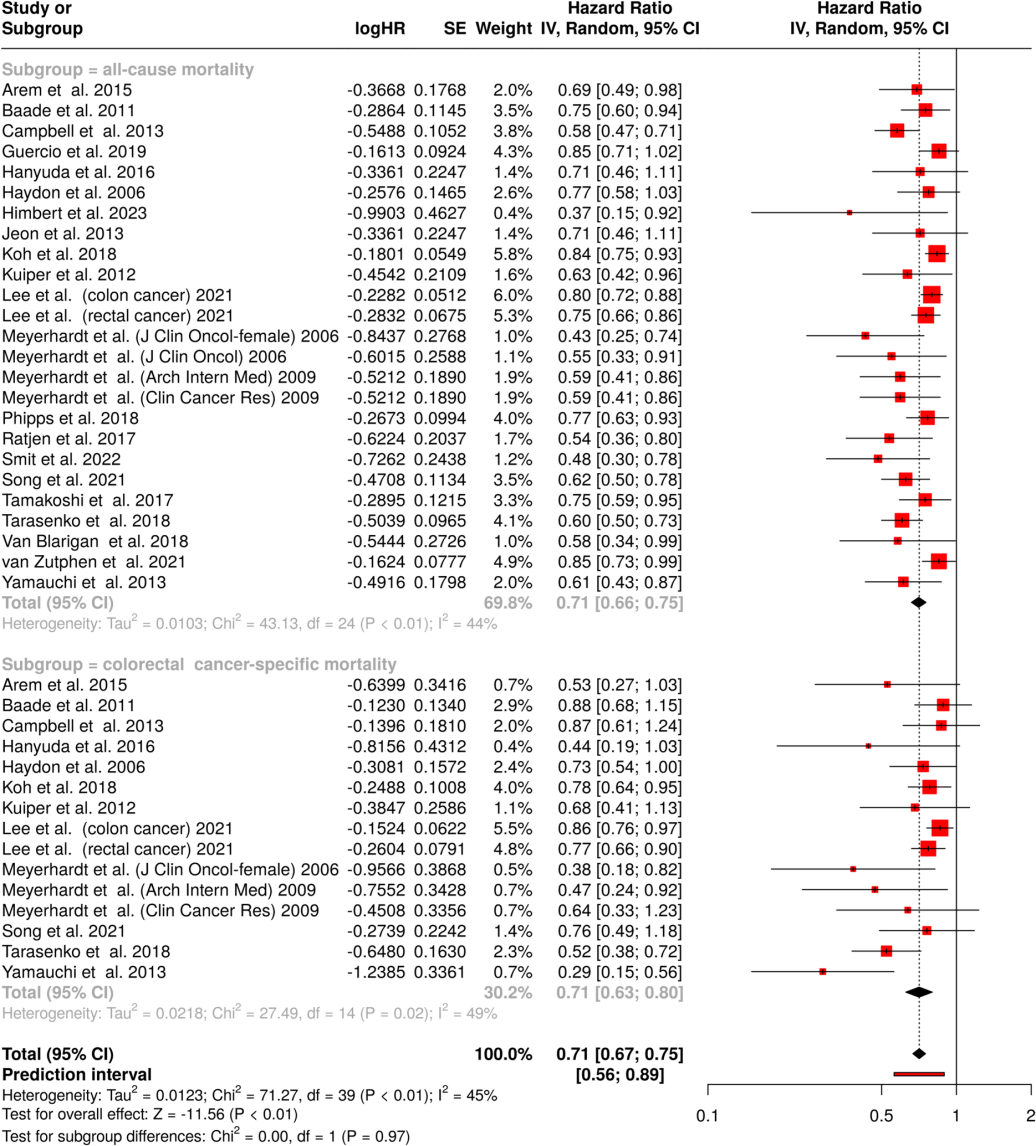
Forest plot showing the association between physical activity and mortality of colorectal cancer patients. The analysis includes two subgroups: all-cause mortality (upper panel, n = 25 studies) and colorectal cancer-specific mortality (lower panel, n = 15 studies). The pooled analysis demonstrates a significant protective effect of physical activity, with a pooled HR of 0.71 (95% CI: 0.67–0.75). Physical activity showed similar beneficial associations on all-cause survival (HR: 0.71, 95% CI: 0.66—0.75) and colorectal cancer-specific survival (HR: 0.71, 95% CI: 0.63—0.80). Moderate heterogeneity was observed across studies (I^2^ = 45%, *p* < 0.01). Abbreviations:* CI*: confidence interval;* HR*: hazard ratio;* IV*: inverse variance;* SE*: standard error

This meta-analysis investigated the impact of physical activity on survival outcomes in colorectal cancer, encompassing 40 cohorts [107–130]. The analysis stratified outcomes into all-cause and colorectal cancer-specific survival (Fig. 6). Analysis of all-cause survival, which included 25 studies, demonstrated that physical activity was associated with a 29% reduction in all-cause mortality, yielding a pooled HR of 0.71 (95% CI: 0.66—0.75). Significant heterogeneity was observed among these studies (*p* < 0.01), with an I^2^ = 44% indicating that approximately half of the inter-study variability arose from true heterogeneity rather than chance. The assessment of physical activity on colorectal cancer-specific survival, incorporating 15 studies, revealed an identical protective effect, with a HR of 0.71 (95% CI: 0.63—0.80). These studies also exhibited significant heterogeneity (*p* = 0.02), though slightly higher than the all-cause analysis, with an I^2^ value of 49%. The combined analysis of both survival outcomes maintained the consistent protective effect, with a pooled HR of 0.71 (95% CI: 0.67—0.75). The prediction interval (0.56—0.89) suggests that future studies investigating this association would likely observe similar protective effects. Notably, we found no significant differences between the two subgroups (Chi^2^ = 0.00, df = 1, *p* = 0.97). The overall analysis demonstrated moderate heterogeneity (I^2^ = 45%, *p* < 0.01).

Publication bias assessment revealed concerning patterns in both survival outcomes. Analysis of all-cause survival cohorts showed significant funnel plot asymmetry (intercept: − 1.92, 95% CI = − 2.66—− 1.17, t = − 5.052, p < 0.001), as depicted in Fig. 3E. Similarly, for colorectal cancer-specific survival, funnel plot asymmetry was confirmed by Egger’s test (intercept: − 1.83, 95% CI: − 2.71—− 0.94, t = − 4.032, *p* = 0.001, Fig. 3F), suggesting potential publication bias in both outcome categories.

### Skin cancer

This meta-analysis included four cohorts assessing the relationship between physical activity and survival outcomes in skin cancer patients [131, 132]. The pooled analysis estimated a 14% reduction in mortality risk among physically active individuals compared to those with lower activity levels (data not shown). However, the pooled HR was 0.86 (95% CI: 0.71—1.05), indicating that the association did not reach statistical significance. Notably, no significant heterogeneity was observed across the studies, suggesting that the effect sizes were consistent in both magnitude and direction despite differences in study design and patient characteristics (I^2^ = 0%, *p* = 0.48). Given the borderline nature of the findings, additional research with larger cohorts is needed to establish a conclusion for skin cancer.

## Discussion

This meta-analysis provides compelling evidence that post-diagnosis physical activity is associated with improved survival outcomes in patients with breast, lung, prostate, colorectal, and skin cancers. Our findings reinforce the growing body of literature supporting the beneficial role of physical activity in oncology and highlight its potential as a modifiable factor in cancer care. Given that these malignancies are age-related diseases [11], the observed survival benefits may be attributed, at least in part, to the anti-aging effects of physical activity at the cellular and molecular levels [25–27].

The biological mechanisms underlying the association between physical activity and improved cancer survival are multifaceted. Physical activity has been shown to regulate key hallmarks of both aging and cancer, including chronic inflammation, oxidative stress, immune function, and genomic instability [25–27]. Exercise reduces systemic inflammation by downregulating pro-inflammatory cytokines such as IL- 6 and TNF-α while promoting anti-inflammatory mediators [33]. This is particularly relevant in cancer, where chronic inflammation is a well-established driver of tumor progression and metastasis [37, 133–135]. Furthermore, exercise enhances mitochondrial function [35] and reduces oxidative stress through upregulation of Nrf2-mediated antioxidative pathways [136–138], both of which are critical in maintaining cellular homeostasis and preventing cancer- and cancer treatment-related metabolic dysfunction and fatigue. By improving mitochondrial efficiency and promoting cellular resilience, physical activity may support better treatment tolerance in cancer patients [139–141]. Additionally, physical activity is known to lower blood glucose levels and improve insulin sensitivity [142], which may indirectly influence tumor growth.

Physical activity is also known to improve immune surveillance, facilitating the detection and elimination of malignant cells [37, 143–145]. Regular exercise has been linked to increased cytotoxic activity of natural killer (NK) cells and enhanced function of tumor-infiltrating lymphocytes, both of which contribute to improved cancer outcomes [37, 143–145]. Additionally, emerging evidence suggests that exercise may modulate epigenetic mechanisms, potentially influencing gene expression patterns related to tumor suppression and aging [146–149]. Given these diverse mechanisms, it is plausible that physical activity exerts its survival benefits by targeting fundamental biological processes that contribute to both cancer progression and aging.

The findings of this study have important clinical implications, reinforcing the need to integrate physical activity recommendations into cancer survivorship care plans [150, 151]. While conventional treatments such as surgery, chemotherapy, radiotherapy, and targeted therapies remain the cornerstone of cancer management, exercise-based interventions offer a complementary strategy that may enhance treatment efficacy and reduce adverse effects. Given the growing evidence base, oncology guidelines should consider incorporating structured exercise programs as part of routine care for cancer survivors.

The magnitude of survival benefits observed across different cancer types suggests that physical activity should be promoted irrespective of tumor site. The greatest benefits were observed in breast and prostate cancer, which may be attributed to the well-documented influence of hormonal and metabolic pathways on these malignancies. However, significant improvements in survival were also evident in lung and colorectal cancer patients, reinforcing the broad applicability of exercise interventions. While the association in skin cancer did not reach statistical significance, this may be due to the limited number of studies available for analysis, warranting further investigation.

While the benefits of physical activity are increasingly evident, its feasibility during cancer treatment remains an important consideration [152]. Exercise capacity may be limited in patients undergoing intensive therapies or experiencing cancer-related fatigue and cachexia. Nevertheless, emerging evidence suggests that even light-to-moderate physical activity—when tailored to an individual’s capacity—can confer benefit. Multidisciplinary approaches involving oncology rehabilitation specialists may help identify and implement safe, personalized exercise plans [152–155]. Until more detailed evidence is available, general guidelines such as those from the American Cancer Society [156] may serve as a practical framework for clinicians and patients.

Despite the robust findings, several challenges remain in translating this evidence into clinical practice. One key limitation is the heterogeneity in how physical activity is defined and measured across studies. Variations in exercise intensity, frequency, and duration may contribute to differences in observed effects, highlighting the need for standardized assessment tools in future research. Future research should also explore the optimal timing, type, and intensity of exercise needed to maximize survival benefits. While most studies focus on moderate-to-vigorous aerobic activity, emerging data suggest that resistance training may offer additional advantages, particularly in mitigating treatment-induced muscle loss and metabolic dysregulation [157–159]. Personalized exercise prescriptions tailored to individual patient characteristics, cancer stage, and treatment regimen may further enhance outcomes [160–165].

In conclusion, this meta-analysis demonstrates that post-diagnosis physical activity is significantly associated with improved survival in patients with major age-related cancers. Given its well-documented anti-aging and tumor-suppressive effects, physical activity represents a promising, non-pharmacological intervention that can complement conventional cancer treatments. The findings underscore the need for integrating structured exercise programs into cancer care and survivorship plans.
